# Do we need a definition of acute heart failure with preserved ejection fraction?

**DOI:** 10.1080/07853890.2021.1968028

**Published:** 2021-08-25

**Authors:** Agnieszka Kapłon-Cieślicka, Lars H. Lund

**Affiliations:** a 1st Chair and Department of Cardiology, Medical University of Warsaw, Warsaw, Poland; b Unit of Cardiology, Department of Medicine, Karolinska Institutet, and Heart and Vascular Theme, Karolinska University Hospital, Stockholm, Sweden

**Keywords:** Heart failure with preserved ejection fraction, heart failure decompensation, diastolic dysfunction, diagnosis

## Abstract

Heart failure with preserved ejection fraction (HFpEF) might soon become the most prevalent type of acute heart failure. Still, despite more than 30 years of research on HFpEF, not only do we lack specific treatment, but also a generally accepted definition of HFpEF. Since 2016, several definitions and algorithms have been proposed for diagnosing both diastolic dysfunction and overt HFpEF. However, all of them focus exclusively on chronic (and not acute) HFpEF. Recent studies showed that acute HFpEF may be overdiagnosed in patients presenting with acute dyspnoea. The aim of our article was to address two questions: (1) why there is a need for specific diagnostic criteria for acute HFpEF, and (2) what such definition of acute HFpEF should encompass.

**KEY MESSAGES:**
- Several scores and algorithms have been proposed for diagnosing chronic heart failure with preserved ejection fraction (HFpEF), however, so far, there is no definition of acute HFpEF.
- Acute HFpEF seems to be overdiagnosed in patients presenting with acute dyspnoea.
- Definition of acute HFpEF should comprise both (1) features of chronic HFpEF and (2) markers of increased left ventricular filling pressures and/or of pulmonary congestion.

## Introduction: heart failure and ejection fraction

According to projected trajectories of the proportion of heart failure (HF) types among patients hospitalised for HF decompensation, HF with preserved ejection fraction (EF) – HFpEF – might soon become the most prevalent type of acute HF [1]. However, in 2021, despite more than 30 years of research and discussion on HFpEF (and its various nomenclatures), we still lack specific diagnostic criteria of its acute exacerbation, let alone specific treatment in either the acute or chronic setting.

In 2019, the PARAGON-HF trial missed its primary endpoint of cardiovascular death and total HF hospitalisations (“hard endpoints”), and in 2020, the PARALLAX trial failed to demonstrate improvement over the control group in functional capacity, and quality of life improved at 4 but not 24 weeks (“soft endpoints”) with sacubitril-valsartan in HFpEF, despite achieving the anticipated reduction in concentrations of natriuretic peptides (NPs) [2,3]. Some hope remains with trials of sodium-glucose cotransporter 2 (SGLT2)-inhibitors (EMPEROR-Preserved [4], DELIVER [ClinicalTrials.gov Identifier: NCT03619213]), especially given the recent results of the SOLOIST-WHF trial [5].

Both the PARAGON-HF and the PARALLAX trial included not only patients with normal EF but also those with “borderline” EF (i.e. with EF ≥45% and >40%, respectively) [2,3]. Earlier, in 2016, the European Society of Cardiology (ESC) guidelines introduced, for the first time, a separate HF type – HF with mid-range EF (HFmrEF), and clearly defined EF cut-offs for HFmrEF (40–49%) and HFpEF (≥50%) [6]. This distinction was made to stimulate research into the underlying pathophysiology and treatment of those “grey zone” patients in order to elucidate whether they are closer to HFpEF or HF with reduced EF (HFrEF). After 5 years of research, it is becoming increasingly clear that HFmrEF, which should more appropriately be termed HF with mildly reduced instead of “mid-range” EF, is milder with better prognosis, but otherwise resembles HFrEF more than HFpEF, and may respond to similar treatments as does HFrEF [7–11]. In contrast, HFpEF, i.e. truly “normal” EF differs from HFrEF and HFmrEF and treatments will need to target specific phenotypes such as transthyretin-related (ATTR) amyloidosis (tafamidis in the ATTR-ACT study) or hypertrophic cardiomyopathy (mavacamten in the EXPLORER-HCM trial) or potentially comorbidity-driven systemic inflammation and microvascular dysfunction [12–15].

## Defining HFpEF

Following the HFpEF definition from the 2016 ESC guidelines, several algorithms were proposed for diagnosing both diastolic dysfunction and overt HFpEF [6,16–18]. The 2018 H_2_FPEF score includes 6 dichotomised, clinical and echocardiographic variables: H1 for “heavy” (body mass index >30 kg/m^2^), H2 for hypertension treated with 2 or more medicines, F for the presence of atrial fibrillation (AF), P for echocardiographic signs of elevated pulmonary artery pressure, E for elderly (age of >60 years), and F for elevated left ventricular (LV) filling pressures on echocardiography (E/e’ ratio >9). If positive, these variables are attributed 1–3 points with a total maximum of 9 points and a high probability of HFpEF at scores of 6–9 [17]. In contrast, the 2019 Heart Failure Association (HFA)-PEFF algorithm is a more complex, stepwise approach with step 1 [P] for pre-test assessment, step 2 [E] for echocardiography and NP score, step 3 [F1] for functional testing (exercise echocardiography and/or heart catheterization), and step 4 [F2] for final aetiology [18]. Compared to the 2016 ESC definition of HFpEF, the 2019 HFA-PEFF algorithm is more specific for HFpEF, and the 2018 H_2_FPEF score has the advantage of validation against invasive measurements [6,17–19]. Still, despite their strengths, both those HFpEF definitions are to some point diagnoses “by exclusion” i.e. they refer to symptomatic patients in whom other potential reasons for exertional dyspnoea have been ruled out. Furthermore, all hitherto HFpEF definitions focus exclusively on chronic (and not acute) HFpEF [6,16–18].

## Acute HFpEF

Diagnosing acute HFpEF relies mostly on clinical judgement. For example, in an elderly patient with acute dyspnoea, classical HFpEF risk factors and comorbidities (obesity, hypertension, diabetes, AF), rales at the basis of the lungs, elevated NPs concentrations, and preserved EF on echocardiography, it is tempting to diagnose HFpEF. However, such patients often have other cardiac and extracardiac comorbidities, which may, at least in part, be responsible for their clinical presentation. Basal rales on lung auscultation are not pathognomonic for pulmonary congestion and may result from inflammation, fibrosis, interstitial lung disease, or atelectasis (the latter often observed in obese and less mobile patients). Concentrations of NPs may rise above the adopted “HF cut-points” in patients with no HF, but with other cardiac (AF, valvular heart disease) and extracardiac (pulmonary embolism, chronic obstructive pulmonary disease, liver disease, renal dysfunction) diseases, especially if they coexist, which is common in the elderly. Older age and lower body mass index are also associated with higher levels of NPs [6,18,19]. Thus, NPs cut-offs for HF adopted in the 2016 ESC guidelines are recommended for excluding, but not for confirming an HF diagnosis [6]. Conversely, the 2018 H_2_FPEF score did not include NPs at all, as their addition to the score did not increase its diagnostic ability [17]. In the 2019 HFA-PEFF algorithm, elevated NPs are not required to establish the diagnosis of HFpEF but increase its likelihood. Different cut-offs are proposed depending on the presence or absence of AF, and on the type of diagnostic criterion (major or minor) [18]. These scores and those cut-offs, however, refer to chronic HFpEF, and cannot be used for diagnosing acute HFpEF.

Thus, there is a need for separate diagnostic criteria for acute HFpEF. Most parameters included in algorithms for the diagnosis of chronic HFpEF or diastolic dysfunction, such as NPs, E/e’ ratio, mitral inflow velocities, tricuspid regurgitation velocity, and to a lesser extent left atrial volume index and e’ velocities, change with volume and pressure overload, as well as heart rate, and thus cut-offs used for chronic HFpEF may not be applicable in patients with acute symptoms [19].

**Figure 1.**
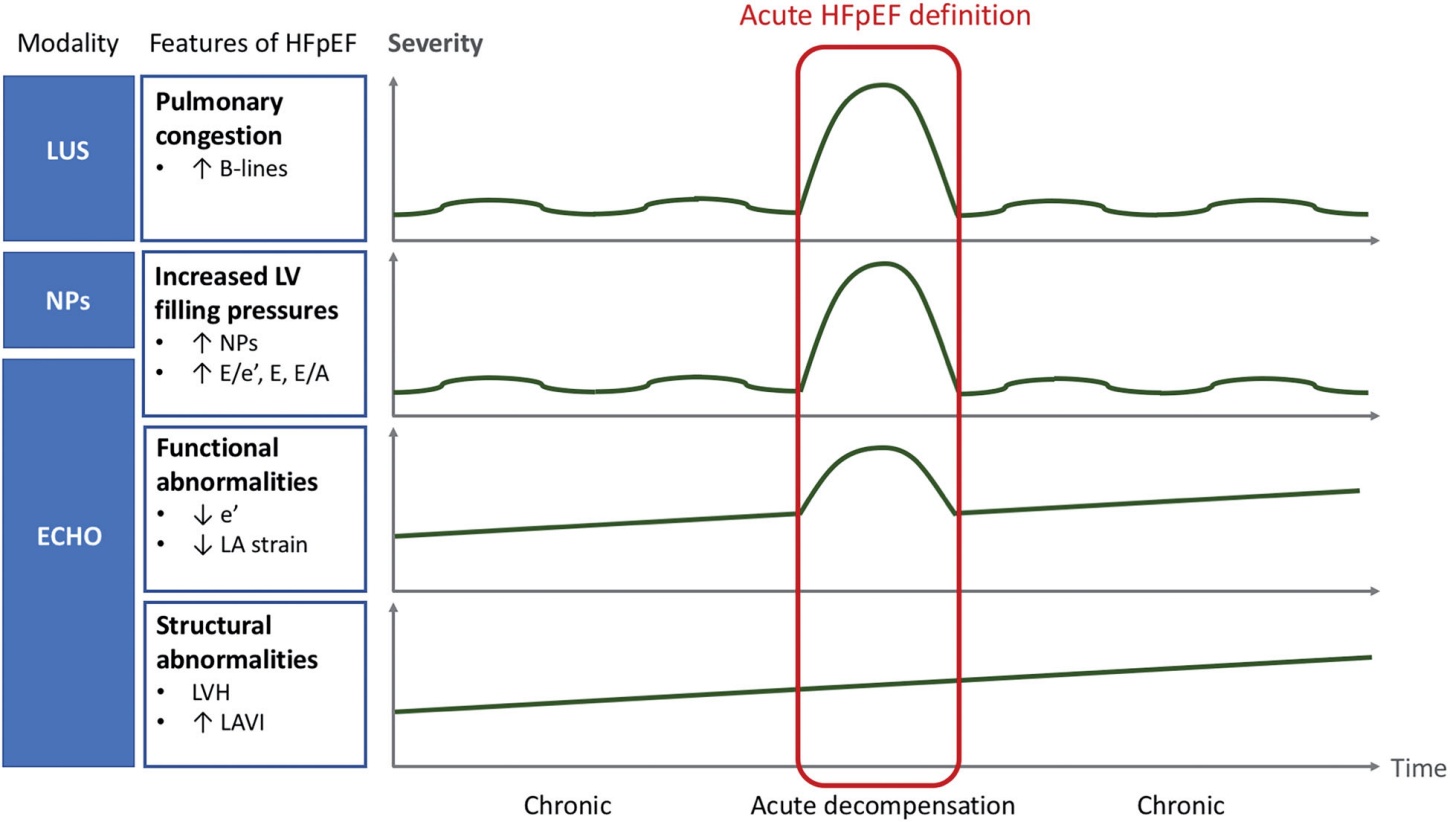
Proposal of an integrated approach to the diagnosis of acute heart failure with preserved ejection fraction (HFpEF) in patients with preserved EF and acute symptoms suggestive of HF. LUS: lung ultrasound; NPs: natriuretic peptides; ECHO: echocardiography; LV: left ventricle; LA: left atrium; LVH: left ventricular hypertrophy; LAVI: left atrial volume index.

In a patient with preserved EF and acute symptoms suggestive of HF, two questions need to be addressed: (1) whether the patient has HFpEF, and (2) whether HFpEF is the reason for present symptom exacerbation. Regarding the first question, of patients hospitalised for acute HFpEF, as many as half might not meet even the relatively mild criteria for chronic HFpEF during the index hospitalisation, and three quarters might not meet chronic HFpEF criteria at stable follow-up [20,21]. Even if a patient does indeed have chronic HFpEF, he or she still may experience acute dyspnoea due to other cardiac or extracardiac causes, unrelated to HFpEF. Cardiac dyspnoea results from pulmonary congestion, which is secondary to the elevation of left atrial pressure (LAP). The latter can be either measured invasively or estimated by echocardiography using E/e’ ratio and mitral inflow velocities [16,22]. In a patient with dyspnoea at rest or with minimal physical activity, normal LAP precludes left-sided cardiac origin of dyspnoea, including HFpEF. Concentrations of NPs correlate with LV filling pressures but have limitations presented above [6,18,19]. The presence of B-lines in lung ultrasound is an easily obtainable and reliable marker of pulmonary congestion, superior to chest X-ray, and proved its diagnostic and prognostic value both in hospitalised and ambulatory patients with HF, including HFpEF [22]. Still, B-lines on lung ultrasound are not pathognomonic for cardiogenic pulmonary congestion but may be present in patients with pneumonia or acute respiratory distress syndrome (ARDS), who will also present with acute dyspnoea [22]. Thus, all those modalities applied together (NPs, echocardiography with LAP estimation, lung ultrasound) help establish the diagnosis of acute HFpEF, and their incorporation in future diagnostic algorithms should be advocated. Still, in a patient with acute dyspnoea and preserved EF, acute HFpEF is not the only possible reason for LAP elevation, as it may also result from the onset of AF, rise in arterial pressure, valvular disease, or volume overload (e.g. due to renal failure) – these conditions can induce LAP elevation each on their own or in combination even in the absence of HFpEF, but they can also trigger chronic HFpEF decompensation. As they are common in elderly patients suspected of HFpEF, acute HFpEF definition should also include indices of chronic HFpEF such as LV hypertrophy, and/or decreased e’ velocities, given that acute HFpEF exacerbation can only develop on top of previous chronic diastolic dysfunction (in contrast to HFrEF which can acutely develop *de novo*, i.e. in an individual with previously normal LV structure and function). In an acute HFpEF definition, cut-offs for NPs should be adopted for acute HFpEF if possible, and preferably adjusted for the presence or absence of AF. A proposed concept of an integrated approach to acute HFpEF definition is graphically presented in Figure 1. Given the limitations of each individual parameter, the definition of acute HFpEF should, ideally, encompass a broad range of indices of chronic structural and/or functional LV abnormalities, and of acutely increased LV filling pressures. These parameters should be interpreted in conjunction with one another to increase the accuracy of acute HFpEF diagnosis.

At the same time, we acknowledge the limitations of such a complex approach in the acute setting. In the emergency department, treatment of pulmonary congestion must often be prioritised and based on clinical judgement before applying more sophisticated diagnostic tests. Such “empirical” treatment, usually consisting of intravenous diuretics and/or vasodilators will lead to a reduction in LAP and, consequently and desirably, an improvement in symptoms. If echocardiography is delayed, then, at the time of assessment, LAP might have already normalised with treatment, hindering the “post-hoc” confirmation of acute HFpEF. However, the use of point-of-care ultrasound is becoming more and more common in emergency departments and intensive care units, and NP concentrations can also be measured within the first few hours of admission. Finally, as emphasised above, features of chronic HFpEF (i.e. of structural and/or functional LV abnormalities), which can be assessed after achieving clinical stabilisation, are obligatory to secure the diagnosis of acute HFpEF decompensation. Typical features of chronic HFpEF are gathered under the functional and morphological domains of the HFA-PEFF score [18]. If LAP elevation and/or pulmonary congestion were not documented in a patient with suspected acute HFpEF decompensation and the result of the HFA-PEFF score (step 2 [E] of the HFA-PEFF algorithm) remains inconclusive, once the patient is stabilised, diastolic exercise test (step 3 [F1] of the HFA-PEFF algorithm) should be considered to confirm HFpEF [18].

Regardless of EF category, when validated externally, HF could be potentially misdiagnosed in 15–50% of hospitalised patients and half of the ambulatory, primary care patients [21,23–29]. This demonstrates the need to objectify HF diagnosis which is still most often made clinically [21,23–29]. The proposed integrated approach, presented in Figure 1, is based on objective evidence from different diagnostic tests in patients with an initial clinical diagnosis of acute HFpEF. While the leading symptoms and signs of HF are less specific (dyspnoea, pulmonary rales, tachycardia, peripheral oedema), some signs (third heart sound, elevated jugular venous pressure, hepatojugular reflux) may be more specific for HF, but are not always present (e.g. third heart sound present in 30%, and elevated jugular venous pressure in 35% of acute HF patients) [11,30]. Importantly, all HF symptoms and signs may occur in patients with volume overload (e.g. in renal failure) and/or high-output states (e.g. anaemia, thyrotoxicosis, arteriovenous shunts, liver disease), even in the absence of an underlying LV disease. Those clinical symptoms and signs can mimic HF but will resolve once the primary cause is appropriately treated [11]. This underscores the need for an accurate assessment of features of chronic LV abnormalities in a patient with a clinical suspicion of acute HFpEF.

Yet, given that there is no evidence-based treatment for acute HFpEF, does it matter if we correctly diagnose it? Diuretics may help resolve symptoms of increased LAP irrespective of its aetiology. However, in patients with normal LAP and no hypervolemia, diuretic use may be detrimental, especially in the elderly with reduced glomerular filtration. Furthermore, overdiagnosing HFpEF may lead to disregarding other potential cardiac or extracardiac causes of symptoms. In patients with preserved EF and a clinical diagnosis of acute HF, compared to patients with increased LAP (and thus “confirmed” acute HFpEF), those with normal LAP (and thus possibly misdiagnosed with HFpEF) were subsequently twice as often hospitalised for non-cardiovascular reasons and significantly more often hospitalised for cardiovascular non-HF reasons, despite a lower prevalence of cardiac and extracardiac comorbidities at baseline [21]. This suggests that at least some patients with acute dyspnoea and preserved EF may be overdiagnosed with HFpEF and underdiagnosed with other diseases, which consequently leads to their exacerbations. This emphasises the need for careful assessment of patients with suspected HFpEF for cardiovascular and non-cardiovascular comorbidities.

A reliable definition of acute HFpEF is needed also because many patients are first diagnosed with HFpEF during hospitalisation for acute symptoms. Once “labeled” with HFpEF, their chronic symptoms such as dyspnoea, fatigue, exercise intolerance or peripheral oedema may later be automatically attributed to HFpEF, delaying the diagnosis of other relevant comorbidities, such as chronic obstructive pulmonary disease, interstitial lung diseases, pulmonary arterial hypertension, venous thromboembolism and chronic thromboembolic pulmonary hypertension, chronic coronary syndromes, chronotropic incompetence, constrictive pericarditis, thyroid dysfunction, kidney disease, liver disease, anaemia, myelodysplastic and myeloproliferative syndromes, systemic autoimmune diseases, neuromuscular disorders, or cancer disease. Given the natural history of HFpEF, the definition of acute HFpEF should comprise both (1) features of chronic HFpEF (such as LV hypertrophy and decreased e’ velocities) and, obligatory, (2) markers of increased LV filling pressures (such as elevated NPs, increased E/e’ ratio, high E-wave velocity with pseudonormal or restrictive mitral inflow pattern) and/or of pulmonary congestion (such as B-lines on lung ultrasound). Almost every chronic disease has a definition of its acute exacerbation, and HFpEF should be no exception.

## Data Availability

Data sharing is not applicable to this article as no new data were created or analysed in this study.
